# Differential Impact of Parental Practices and Parental Emotional Clarity on Child Symptoms in Single-Child vs. Multiple-Child Divorced Families

**DOI:** 10.3390/children11121481

**Published:** 2024-12-03

**Authors:** Laura Merino, Ana Martínez-Pampliega, Marta Herrero

**Affiliations:** Department of Psychology, Health Sciences Faculty, University of Deusto, 48007 Bilbao, Spain; martinez.pampliega@deusto.es (A.M.-P.); m.herrero@deusto.es (M.H.)

**Keywords:** sibship size, divorce, child symptomatology, parenting

## Abstract

Objective: The main objective of this study was to analyze the differences in parental emotional clarity and parental practices among families with a single child and families with more than one child, and their relationship with the children’s internalizing and externalizing symptomatology, specifically, anxiety–depression and aggressive behavior in a conflictive divorce context. Methods: The participants were 247 Spanish divorced parents. In total, 62% of the participants reported being the parents of one child and 38% of two children. All participants answered questionnaires that measured the variables investigated in this study. Results: The results supported the working hypothesis that families with more than one child present with less emotional clarity, which, concatenated with critical and rigid parental guidelines, is associated with children’s greater presence of anxious–depressive and aggressive symptoms. Conclusions: Families with more than one child have less positive parental guidelines and so their children express more symptoms.

## 1. Introduction

Although numerous population and sociological investigations have focused mainly on the relationship between family size and children’s well-being [1,2], there are hardly any studies from psychology that analyze in-depth the mediating processes that explain the impact of family size, specifically sibship size, on the aspects of family functioning, such as parental practices and parental emotion regulation. Most of these studies are based on theoretical models that propose solid hypotheses about the relationship between sibship size and the children’s well-being; however, empirical studies in this area are very limited [3]. The few existing empirical studies have primarily addressed children’s well-being. Moreover, the comprehension of these family variables (e.g., sibship size) is especially relevant in the field of conflictive divorce, where parenthood functions act as an essential mediating variable regarding the impact of divorce on children’s well-being. Therefore, deepening the analysis of the processes involved in children’s well-being, and not only focusing on interparental conflict, would allow us to progress our knowledge in this area.

### 1.1. Sibship Size and Children’s Well-Being

On the one hand, previous research has shown that sibship size is positively associated with behavioral disorders and delinquency risk, such that having more siblings facilitates the presentation of more symptoms of behavior disorders [4,5] due to less parental supervision in families with several children [6]. On the other hand, sibship size is a consistent predictor of academic performance, as the fewer siblings there are in a family, the better the children’s academic performance, as already proven by Phillips [7] with data collected in different regions of the world (the United States, Europe, and Asia).

In an attempt to find possible explanations about the impact of sibship size on the consequences for children from a sociological perspective, several authors proposed the resource dilution explanation [8,9,10], which can be described as “the more children, the more these resources are divided and hence, the lower the quality of the output” [8]. That is, the parental resources available to a family, both material and emotional, are limited. Therefore, the more children a family has, the fewer resources that are available to each child. This is true not only regarding the number of children, but also in times of high family stress such as conflictive divorces: due to the higher demands from a high interparental conflict context, there are fewer parenting resources available to face the challenges of that stressful situation. As a result, high-conflict divorced parents could be emotionally overwhelmed and unable to meet the demands of parenting if they are investing a great amount of effort, time, and emotional resources in the conflict with their ex-partner. This overwhelming situation, that implies less available resources for parents, could be related to parents’ poor emotion regulation strategies.

This approach coincides with what was highlighted by another more recent study, which showed the difficulty that parents of several children have in devoting sufficient attention to them as well as monitoring their behavior. As a result of this situation, the children’s well-being may be harmed [11]. However, on the contrary, some studies also identify certain mental health benefits for children who have siblings because of the support and companionship they provide [12,13]. These contradictory results require a greater understanding of the mechanisms through which family context variables, such as sibship size, impact children’s emotional development.

### 1.2. Parenthood as a Mediating Variable Between Sibship Size and Children’s Well-Being

As recent literature about parenting has highlighted [14], there are different possible approaches to understanding parenthood that include different parenting dimensions. In order to gain theoretical coherence, this stud

y is grounded in Morris et al.’s [15] theoretical model of parenting because it emphasizes the emotional variables of parenting that are relevant to the aim of this study.

In their review of the literature, Morris et al. [15] proposed the tripartite model to explain the impact of the emotional family context on children’s emotional development, which takes place through three pathways: (1) the emotion regulation of the parents themselves, (2) parental practices, and (3) the emotional family climate.

First, concerning the first pathway focused on the emotion regulation of parents, it has been proven that parents are direct and indirect models in the comprehension and communication of emotions for their children [16]. In the field of emotion regulation of parents, emotional clarity emerges as one of the central aspects [17], and is understood as the ability to identify, recognize, discriminate, and understand one’s emotions [18]. Emotional clarity is identified as a phase that comes prior to any process of emotional modeling from parents to children (for reviews see [19,20]). Research has shown that emotional clarity facilitates adaptive strategies for emotion regulation [21]. Additionally, on the contrary, deficits in emotional clarity are associated with less adequate coping responses [22], hindering an adaptive and effective response to stressful interpersonal situations [18]. Regarding children’s outcomes from the emotion regulation process of their parents, there are several studies that support the mediating role of the parents’ emotional clarity between family functioning and the children’s depressive symptoms [22,23,24,25].

Second, and following the second pathway indicated by Morris et al. [15], children’s well-being is also strongly influenced by parental practices [26,27,28]. On the one hand, the literature shows the negative impact of authoritarian parental practices on children’s well-being. This style is characterized by low warmth, low responsiveness to children’s needs, and high levels of coercive control or rigid discipline [26]. In particular, the authoritarian parenting practice seems to be associated with negative coping strategies and aggressive behaviors in the children [29,30]. Regarding children’s outcomes from the emotion regulation process of parents and within the authoritarian parenting practice, it is precisely the aspect of rigid or coercive discipline that has shown an association with the aggressiveness displayed by children [31,32]. Rigid parental practices are associated with harsh, insensitive, and unresponsive parental practices. Rigidity has been associated with low parental competence in vulnerable populations and is one of the most potent parental predictors of child abuse and neglect [33]. On this topic, one study [34] hypothesized that rigid parental practices can turn children’s behaviors into predictable maladaptive patterns that include depressive symptoms and aggressive behavior. However, other studies indicate bidirectional processes in the association of negative parental practices (including rigidity, excessive control, and hostility) and children’s behavioral disorders [35]. These results are usually better understood from the theory of coercive cycles of Patterson [36], according to which coercive interactions between parents and aggressive children escalate over time and this contributes to the development of children’s antisocial behavior. In addition, authoritarian parenting practices have also been consistently linked to infant–juvenile anxiety in the literature, associated with higher levels of anxiety or other internalizing symptoms [37]. A review of the recent literature [38] found consistent evidence linking parents’ affection, warmth, and acceptance with lower levels of anxiety or other internalizing symptoms in their children. Also, anxiety in children is associated with parental rejection, psychological control, over-involvement, excessive control, rigid discipline, and hostile control. These findings coincide with the approaches of cognitive models of anxiety, according to which excessive control and lack of warmth can transmit a perception of generalized threat and a feeling of personal inefficiency to children, which explains the onset and development of anxiety.

Third, and concerning the third pathway proposed by Morris et al. [15], the emotional family climate is also an important aspect of parental practices that influences children’s well-being. Expressed emotion (EE) is a measure of the family’s emotional climate and a robust predictor of several disorders, including depression (for a review, see [39,40]). Specifically, the “criticism” component of EE is the most potent predictor of depression in children and adolescents (e.g., [41,42,43]). On this topic, numerous articles have highlighted that the parents of children and adolescents with depressive symptoms presented parental practices characterized by low levels of affection, support, and communication, and high levels of criticism [39,40,44] and conflict [40,45]. There are several situations where the impact of the family climate significantly affects the children’s well-being, as in the case of conflictive divorced families. The study of the relationship between the emotional family climate and children’s well-being is especially relevant in those situations in which parenthood is particularly affected, as in the case of divorced families [46,47].

Additionally, several studies have shown that the alteration of different aspects of parenthood associated with interparental conflict is the essential mechanism for understanding children’s well-being in conflictive divorced families [48,49]. Studies show that conflictive divorced parents exercise a more inconsistent discipline, with a lower level of affection and less emotional availability for their children [50,51]. Therefore, the children of these divorced families are more negatively affected by their parents’ parental practices and emotion regulation difficulties, and this is associated with greater emotional, behavioral, and social problems [51].

Finally, there is literature-based evidence that the addition of more children to the family introduces further stress and complexities to family life. Although the impact of the demands related to parenting multiple siblings has not been thoroughly studied, it is likely that parents who struggle more with these demands or experience higher stress levels may be less effective in their parenting [52], specifically in their ability to emotionally regulate themselves [53]. For example, they may have more confusion about their own feelings, which matches the lack of emotional clarity included in the first pathway of Morris’ theoretical model.

To sum up, a better understanding of children’s well-being in the context of conflictive divorce and in terms of the differential impact of emotional clarity and parental practices, and taking sibship size into account, would be an important step forward in this area. To date, however, there have been no studies in this direction.

### 1.3. The Present Study

A review of the literature reveals the contradictions about the understanding of the impact of sibship size on children’s psychological well-being. The limited progress in the comprehension of the mediating mechanisms that could help us understand these differences and the lack of studies in the context of parental divorce has also been revealed.

Therefore, this study aims to cross-sectionally analyze the role of sibship size, focusing on the context of divorce and following the model of Morris et al. [15]. That is, the role of parental emotional clarity and its relationship with parental practices will be analyzed to explain children’s internalizing and externalizing symptomatology, specifically, anxiety–depression and aggressive behavior. The extent to which sibship size conditions this relationship will also be analyzed. Specifically, the main hypothesis is that the lack of parental emotional clarity will be related to children’s symptomatology through authoritarian parental practices, and this relationship will be moderated by sibship size. We expect that families with more than one child will present a greater lack of emotional clarity.

## 2. Method

### 2.1. Participants

The participants were 247 Spanish parents who use family visitation centers. All parents were divorced but there were no ex-partners in the sample. The average age of the participants was 40.95 years (SD = 6.39); 42% were men and 58% were women. Concerning the level of education, the participants reported having completed primary studies (33%) or high school (36%). They were less likely to report having medium careers (12%), completing higher studies (13%), having a master’s degree/doctorate (5%), or not having attained primary school (<1%). Most parents (48%) had been separated/divorced for more than three years; 13% from two to three years; 21% from one to two years; 10% from six months to one year; 7% from two to six months; and 1% for less than two months.

Concerning sibship size, 62% of the participants reported being the parents of one child and 38% of two children. Regarding custody, 30% of them were in joint physical custody and 70% were in joint legal custody. The mean age of only children was 7.03 years (SD = 3.74); 50% were boys and 50% were girls. In the case of two children, the mean age of the oldest child was 11.68 years (SD = 4.53) and that of the youngest was 7.83 years (SD = 4.22). The older children were boys in 44% of the cases and girls in 56%. Among the younger children, 49% were boys and 51% were girls.

### 2.2. Instruments

The sociodemographic variables, such as type of custody as well as the number and age of the children, were collected through an ad hoc questionnaire along with other descriptive variables of the participants. Interparental conflict (IPC) was assumed because all participants were referred to family visitation centers by judicial order due to their high levels of interparental conflict.

### 2.3. Symptom Variables in the Children

Anxiety/depression. For this purpose, we used the “Anxiety/Depression” subscale of the Child Behavior Checklist (CBCL; [54]) in its Spanish version adapted by García et al. [55]. This instrument evaluates the presence of symptoms of agitation, loneliness, or fear in children in the last 6 months. Parents report these symptoms in their children. Examples of the items are “Seems sad for no apparent reason” or “Is nervous or tense”. The items are rated on a 3-point scale: (0 = Not true; 1 = Somewhat true, sometimes true; 2 = Very often or quite often true). In the subsequent analyses, the raw scores of the variable were used. The mean reliability values in this study were adequate in both the results for older children (α = 0.82, ω = 0.83) and younger children (α = 0.69, ω = 0.71). The validity of this instrument has been shown in a large number of works [56].

Aggressiveness. Like anxiety/depression, this variable was measured with the “Aggressiveness” subscale of the Child Behavior Checklist (CBCL; [54]). In this subscale, parents are asked how often their children get into fights, break objects, are disobedient, and show other symptoms of aggression. Examples of items are “Breaks family members’ or other children’s belongings”, “Hits other people”, or “Is disobedient”. In the subsequent analyses, the raw scores of the variable were used. The average reliability was high both for older children (α = 0.92, ω = 0.92) and younger children (α = 0.90, ω = 0.91).

### 2.4. Emotion Regulation Variable

Lack of emotional clarity. Emotion regulation was measured with the Difficulties in Emotion Regulation Scale (DERS; [17]) using the Spanish adaptation by Hervás and Jódar [57]. This scale examines the difficulties that may emerge in the process of emotion regulation and consists of 25 Likert-type items, grouped in five dimensions: non-acceptance, lack of objectives, impulsiveness, lack of strategies, and lack of clarity. It also has a global score for emotion regulation. In this study, only the “Lack of Clarity” subscale was used (e.g., “I have a hard time understanding why I feel the way I feel”; “I am confused about how I am feeling”). This subscale has adequate reliability (α = 0.64, ω = 0.78).

### 2.5. Parental Practices Variable

Criticism. For the study of this variable, the “Criticism” subscale of the Escala de Afecto-Parent Version (EA-P [Scale of Affection]; [58]) was used, which evaluates parents’ criticism, rejection, and lack of confidence toward their children. Examples of items on this scale are “What he/she does is wrong” or “I wish my child were different”. The subscale consists of 10 items, which are rated on a 5-point Likert scale ranging from 1 (never) to 5 (always). The reliability of this subscale is high (α = 0.75, ω = 0.75).

### 2.6. Emotional Family Climate Variable

For the study of this variable, we used the “*Escala de Normas y Exigencias*”-*parent version* (ENE-P [Scale of Rules and Requirements]; [58]), which investigates how parents establish and demand compliance with their rules. In this study, only one of the “Rigid Form” subscales was considered, which includes parents’ imposition of the rules and the high or inadequate demand to follow them (e.g., “I tell him/her that I’m the boss at home”). The scale consists of 8 items, which are rated on a 5-point Likert scale ranging from 1 (*never*) to 5 (*always*). The reliability of this subscale is high (α = 0.74, ω = 0.73).

### 2.7. Procedure

This study was carried out through the National Federation of Family Visitation Centers (Spain). Family visitation centers from 17 different regions of Spain were invited to participate and the sample was recruited among the family visitation centers from the 12 regions that agreed to participate. The managers of the family visitation centers informed parents about the aim of the study, and they highlighted that their participation was voluntary and confidential. The parents who consented to participate completed the questionnaires individually in about 30 minutes. Informed consent was obtained from all subjects involved in the study. Having a diagnosis of severe psychopathological disorders or the existence of domestic violence were exclusion criteria, and these criteria were checked through the judicial referral report and/or the case history registered at the center. Data were collected during the years of 2016–2017, and the study was approved by the Ethics Committee of the University of Deusto (ETK-7/16-17). All procedures contributing to this work complied with the original Declaration of Helsinki. Data are available upon request of the authors.

### 2.8. Analysis Strategy

In our preliminary analyses, possible confounding variables were examined. The relationships between the number of children with the gender of the parents, the education of the parents, time since the divorce, and the gender of the children were explored. The chi-square differences computed by contingency tables showed that none of the sociodemographic variables were related to the number of children, the gender of the parents (χ^2^(1) = 0.05, *p* = 0.831), the education of the parents (χ^2^(5) = 6.03, *p* = 0.303), time since the divorce (χ^2^(6) = 9.72, *p* = 0.137), or the gender of the children (χ^2^(1) = 0.57, *p* = 0.450).

As well, the relationships between those four sociodemographic variables (i.e., gender of the parents, education of the parents, time since the divorce, and gender of the children) and the psychological variables of study (i.e., lack of emotional clarity, rigid parental patterns, critical affectivity, and children’s symptomatology) were examined by the Student’s *t*-test for independent samples. Of these comparisons, only four were significant. Concretely, time since divorce and education of the parents were not related to any of the psychological variables. Gender of the children was only related to children’s aggressiveness (*t* = 3.30, *p* = 0.001), with boys (M = 0.58, SD = 0.41) displaying significantly higher levels than girls (M = 0.39, SD = 0.31). Gender of the parents was related to children’s aggressiveness (*t* = −3.42, *p* = 0.001) and anxiety (*t* = −3.28, *p* = 0.001) and to critical affectivity (*t* = −2.36, *p* = 0.019). In these cases, mothers (M = 15.97, SD = 4.45) showed higher levels of critical affectivity than fathers (M = 14.71, SD = 3.62), and they reported higher levels of aggressiveness in children (M = 0.56, SD = 0.41) and anxiety (M = 0.50, SD = 0.35) than men (aggressiveness: M = 0.40, SD = 0.32; anxiety: M = 0.36, SD = 0.31).

In our main analyses, we first analyzed the differences between the group of parents with one or two children regarding lack of emotional clarity, rigid parental patterns, critical affectivity, and children’s symptomatology through comparisons of means using the Student’s *t*-test for independent samples. To assess the effect size, Cohen’s *d* was calculated, where values of 0.20 indicated small effect sizes, 0.50 medium effect sizes, and 0.80 large effect sizes [56].

Second, we analyzed the correlations between the quantitative variables under study, considering *r*-values of about 0.10 as small correlations, around 0.30 as medium correlations, and 0.50 as large correlations [56].

**Figure 1:**
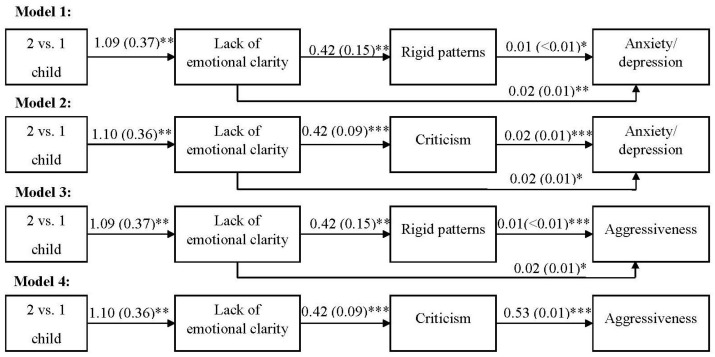
Significant beta coefficients and standard errors of the mediation models. * *p* < 0.05, ** *p* < 0.01, *** *p* < 0.001.

Third, mediation analyses were carried out using the PROCESS 3.27 program (model 6) [59] with a bootstrap of 5000 samples and 95% confidence intervals (CI) to test the hypotheses. In total, four sequential models were tested, two for each dependent variable (i.e., anxiety/depression and aggressiveness of the children), and included the number of children as an independent variable (two children vs. one child), the lack of emotional clarity as the first mediator, and rigid parenthood (Model 1 and Model 3) or critical affectivity (Model 2 and Model 4) as the second mediator (see Figure 1). To assess the effect size of these models, we used Cohen’s [56] criteria to interpret *r*^2^. Specifically, values of 0.01 were considered small, 0.09 medium, and 0.25 large.

## 3. Results

First, the results of the mean differences between the parents with one child versus two children (see Table 1) indicated that parents with two children showed a greater lack of emotional clarity, and more rigid patterns and criticism than parents with one child. However, there were no differences between the two groups in the children’s symptomatology. The effect sizes of the significant differences were small–medium.

Second, the correlations (see Table 2) between the quantitative variables studied indicated that the lack of emotional clarity and rigid patterns were positively and significantly related to children’s anxiety/depression and aggressiveness, with small correlations. The variable “criticism” was also positively and significantly related to children’s anxiety/depression and aggressiveness, with a small and medium correlation, respectively.

Third, the results of serial mediations tested with PROCESS v3.0 indicated that having two children versus having only one child was associated with a greater lack of emotional clarity in all models (see Figure 1). Also, a greater lack of emotional clarity was related to worse parenting, as it showed positive and significant regression coefficients with rigid patterns and criticism in the respective models. Finally, in the series of effects, both rigid patterns and criticism were related to children’s greater anxiety/depression and aggressiveness in the respective models. In addition to these concatenated effects, a greater lack of emotional clarity was directly related to more anxiety/depression and aggressiveness of the children in all the models except for aggressiveness, when it was predicted by clarity and criticism (Model 4, see Figure 1).

Finally, the mediation analyses of these models indicated that having two children versus having one child was indirectly associated with children’s greater anxiety/depression and aggressiveness. That is, having two children versus having one child was associated with greater aggressiveness in children (Model 3: β = 0.025, SE = 0.013, bootstrap 95% CI [0.005, 0.053]) and anxiety/depression (Model 1: β = 0.025, SE = 0.013, bootstrap 95% CI [0.005, 0.053]); Model 2 (β = 0.020, SE = 0.011, bootstrap 95% CI [0.001, 0.004]) through the greater lack of emotional clarity of the former group. Likewise, it was also observed that the relationship between the greater lack of clarity of the group with two children and anxiety/depression was mediated by rigid patterns (Model 1: β = 0.004, SE = 0.002, bootstrap 95% CI [0.005, 0.053]) and criticism (Model 2: β = 0.009, SE = 0.004, bootstrap 95% CI [0.002, 0.019]).

The same can be observed concerning aggressiveness, both concerning rigid patterns and criticism (Model 3 and Model 4). Thus, the greater lack of emotional clarity of the parents with two children was also related to the children’s greater aggressiveness due to the higher levels of rigid patterns (Model 3: β = 0.004, SE = 0.002, bootstrap 95% CI [<0.001, 0.010]) and criticism (Model 4: β = 0.025, SE = 0.010, bootstrap 95% CI [0.008, 0.046]).

As observed from the *r*^2^ values (Figure 1), these models had a medium effect size, explaining approximately 9% of the variance of the children’s anxiety/depression, and a large effect size regarding the children’s aggressiveness (33% explained variance observed in Model 4).

## 4. Discussion

The main objective of this study was to analyze the role of parental emotional clarity and its relationship with parental practices and emotional family climate to explain children’s internalizing and externalizing symptomatology, specifically, anxiety–depression and aggressive behavior for judicial referral in high-conflict family situations, while also considering sibship size. The results supported the working hypothesis that families with more than one child present less emotional clarity, which, together with critical and rigid parental guidelines, is associated with children’s greater presence of anxious–depressive and aggressive symptoms.

First, the results of this study seem to show that, in conflictive families with several children, there is less emotional clarity, more rigid patterns, and greater criticism than in families with only one child, coinciding with the resource dilution model [8,9,10]. That is, our results are compatible with the fact that parents of two children, compared with parents of a single child, must distribute their emotional parental resources, which leads to more difficulties in emotion regulation (greater lack of emotional clarity) and greater presence of criticism and rigid patterns in their parental practices. However, despite this greater difficulty experienced by multiple-child families, the children of these families, compared with single-child families, do not present with higher levels of internalizing or externalizing symptoms. Accordingly, recent literature [60] has pointed out that sibling relationships can buffer the impact of family functioning on the adjustment of children, and this moderating effect is greater in families with a high level of family stress, such as high-conflict divorced families. However, other studies show a generalization effect of the interparental conflict to more authoritarian and restrictive parental practice that in turn are linked to a decrease in positive sibling exchanges; this is negatively associated with child adaptation [61]. The scientific literature has revealed more complex and new generalization processes with respect to interparental conflict, parenting variables, and sibling relationships in divorced families, and this should be an area of future research.

Second, in this study, these results have been clarified through the use of mediation models. These models have provided evidence that there are differences in the well-being of children of multiple-child families versus the children of single-child families only if parental patterns are taken into account as a mediating variable. The data seem to show, forcefully, that parents in conflictive families with two children have less emotional clarity, which in turn is associated with greater rigidity and criticism in their parental patterns. This concatenation could provoke a greater presence of anxious–depressive and aggressive symptoms in the children. Therefore, the central hypothesis of this study is confirmed and could be explained by the resource dilution model [8,9,10]. However, these results contribute to updating the resource dilution model from a perspective that delves into psychological variables, especially relevant in contexts of parental divorce. That is, from these results, it could be intuited that divorced parents in conflictive contexts who have several children have more difficulty devoting enough attention to monitor the behavior of their children than divorced parents would with a single child. This situation can lead to the harming of children’s emotional well-being, as suggested by the study by Hjern et al. [11].

Third, the aggressiveness displayed by children seems to be an aspect especially affected by sibship size and parental patterns (emotional clarity and parental criticism). In this way, the “lack of emotional clarity” and “parental criticism” variables seem to play an important role in explaining the onset of aggressive symptoms in children, which would need future research for further exploration. In addition, it is relevant to note that, to date, lack of emotional clarity has been identified only as a consistent predictor of children’s depressive symptoms [22,23,24] and not of their aggressive symptoms.

Regarding the limitations of this study, we highlight two of them. On the one hand, the need to homogenize data collection led to parents providing the information, and this makes the results subject to their unique perspective. On the other hand, the cross-sectional design of the research makes it impossible to draw conclusions based on causal effects. Moreover, some studies on parental criticism emphasize the need to work with a latent class membership exhibited during four assessments over 6 months [62]. Therefore, it would be necessary for future research to use longitudinal designs with multi-informants to ensure the robustness of the results. In addition, future research could extend the sample to parents who have experienced a non-conflictive divorce or to non-divorced parents with high interparental conflict in order to extrapolate these results to other family structures.

However, despite these limitations, the study has yielded significant results that help understand children’s symptomatology in situations of parental stress, taking into account the size of the family. This study should extend its results by integrating other variables that to date have been significant and that could contribute to developing this line of research. We emphasize the importance of continuing this study by analyzing the moderation of the gender of children [63,64,65] or of parents [66,67,68,69]. Likewise, we highlight the need to consider the emotion regulation of children themselves as a mediator between the parents’ emotion regulation skills and the children’s symptoms [70]. Even other personal aspects of children could be considered, such as self-esteem, negative cognitive style [71], or personality predispositions [38], which could be affecting the association between parenting and the children’s well-being. Without a doubt, an area of study that should be emphasized is the analysis of the socio-economic level of the family and the economic–social context in which the family operates. In addition, the negative impact of the resource dilution explanation [8] on children’s well-being in families with several children is clearly buffered by the country’s level of development and the available social resources that the welfare state can provide to its population [72].

In summary, the results of this study have highlighted the importance of family factors in understanding the onset of symptoms in children, especially those aspects linked to parental practices and emotional management within the family in situations of high emotional intensity, such as conflictive divorce. Therefore, interventions focused on parent–child dynamics can be useful to prevent and relieve children’s symptoms [73]. Specifically, interventions that enhance communication between parents and children (e.g., [74]) and emotion regulation in both parents and children (e.g., [75]) can be effective in reducing children’s symptoms of discomfort, such as depression or aggressive behaviors.

## Figures and Tables

**Table 1:** Descriptive statistics and comparisons between families with one or two children.

	Total (*n* = 247)		One Child (*n* = 153)		Two Children (*n* = 94)			
Variable	M	SD	M	SD	M	SD	*t*	*d*
Lack of emotional clarity	7.00	2.82	6.58	2.55	7.68	3.10	−3.04 **	0.39
Rigid patterns	23.59	6.55	22.78	6.76	24.89	6.00	−2.48 *	0.33
Criticism	15.44	4.16	14.90	3.70	16.31	4.71	−2.48 *	0.33
Anxiety/depression of children	0.44	0.34	0.43	0.34	0.45	0.34	−0.56	0.06
Aggressiveness of children	0.49	0.38	0.48	0.37	0.51	0.40	−0.68	0.08

* *p* < 0.05, ** *p* < 0.01.

**Table 2:** Correlations between the quantitative variables under study.

	Correlations			
Variable	1	2	3	4
1. Lack of emotional clarity	__			
2. Rigid patterns	0.21 **	__		
3. Criticism	0.31 ***	0.35 ***	__	
4. Anxiety/depression of children	0.21 **	0.20 **	0.27 ***	__
5. Aggressiveness of children	0.19 **	0.24 ***	0.57 ***	0.62 ***

** *p* < 0.01, *** *p* < 0.001.

## Data Availability

The data presented in this study are available upon request of the corresponding author due to privacy issues of the data. The data were collected from centers where the participants are immersed in complicated legal situations such as high-conflict divorces, and although data are anonymous, we are not authorized to share the data.
